# Empowering scientific performance and health: MERIT – A survey study and research perspective

**DOI:** 10.1371/journal.pone.0361623

**Published:** 2026-10-30

**Authors:** Cornelia Herbert

**Affiliations:** Applied Emotion and Motivation Psychology, Institute of Psychology and Education, Faculty of Engineering, Computer Science, and Psychology, Ulm University, Ulm, Germany; Johnson & Johnson MedTech, UNITED STATES OF AMERICA

## Abstract

Academic performance involves achieving one’s goals and reaching one’s full potential while staying mentally, emotionally, and physically well. This can be challenging across all career paths. This article presents results from a new survey examining key psychological factors and how early-career researchers evaluate them in relation to their scientific performance and health as they pursue their career goals. A total of 475 participants (e.g., in Germany, Switzerland, or Austria) evaluated psychological factors that, according to previous research and psychological theories, influence academic success. The evaluations included participants’ assessments of their self-competence, motivation, and work effort; achievement emotions (including negative and positive experiences that influence well-being); perceptions of supervision; and views on reasons for staying or leaving academia. Gender differences and differences between PhDs and postdocs were also analyzed. Overall, PhD students and postdocs felt confident in and satisfied with their academic performance. They evaluated their competencies, effort, and motivation as key to achieving their career goals. Satisfaction, effort, and self-efficacy showed no gender differences, but women reported higher levels of worry and negative emotions, particularly among postdocs. Challenges such as time and financial constraints, as well as the benefits of working outside academia, influenced self-perceptions of career decisions. Most participants reported regular supervision; a few had experience with or were participating in early-career initiatives. The study emphasizes the need to consider young scientists’ beliefs, feelings, and expectations about their academic performance, achievement, and health when developing early-career strategies to reduce perceived barriers and reflect career decisions. The current approach and results are discussed in relation to previous studies, the observed challenges, and the gaps this research addresses.

## 1. Introduction

Universities and research organizations employ a variety of career development tools to support the academic success of early-career researchers [e.g., 1–4]. The need for a healthy academic environment, together with the growing demand for top-tier research programs and scientific excellence, raises questions about which concepts, career tools, programs, and initiatives most effectively foster optimal and healthy peak performance among academics. To help researchers at all stages of their careers realize their full potential, build resilience, and acquire the critical skills needed for academic performance, it is important to examine the psychological factors that foster both healthy and optimal performance, as well as young career candidates’ views on these factors. Based on the results of these investigations, one could establish scientific conceptual frameworks to guide decisions about what scientists, individually and as research teams, need to do to perform at their best. This article presents results from a survey examining key psychological factors relevant to academic performance. It explores how early-career researchers evaluate their own scientific performance, their skills in achieving their academic performance and managing their health, their perceptions of supervision and experience with career tools, as well as their views of daily work barriers and decisions about academic career paths. In the following sections, a comprehensive overview of previous findings and theories guiding the current study will be presented, including the survey’s aim and scope, and the conceptual approaches that integrate performance and health perspectives.

### 1.1 Academic performance: Definitions and theories

A fundamental aspect of scientific work is achieving peak academic performance. The motivation is to generate groundbreaking scientific knowledge that drives scientific advancement and exerts long-term influence on society at large. This can have an impact on the overall scientific reputation of researchers, their academic institutions, and the scientific community, both nationally and internationally [5]. This places high demands on each individual’s capacity to pursue extraordinary scientific achievements, with respect to one’s own career goals, the research team’s goals, and the scientific projects in which one’s activities are embedded. Theoretically, academic performance can be compared to elite sports: achieving peak performance under optimal conditions, including individuals’’ abilities, skills, motivation, and a persistent willingness to improve [6]. Consequently, definitions of peak performance, whether physical or mental, assume that individuals can operate at exceptional levels and sustain above-average results over an extended period [7]. These views and definitions include the implicit assumptions that an individual possesses the ability, motivation, effort, and willingness to continually develop and enhance their performance, skills, actions, ambitions, and interests throughout their academic career. In scientific research, many theoretical, practical, and empirical methods are used to study academic performance [8–11]. These include psychological theories that explain the determinants of peak performance and the factors required to achieve it (see Table 1 for an overview). As summarized in Table 1, although each theory has a specific focus and overlapping or complementary perspectives, there is consensus that academic performance is influenced by multiple factors rather than a single determinant. The factors encompass individual skills and competencies, as well as environmental or systemic conditions, that can influence academic performance either independently or through interactive effects. However, as mentioned above, in academia, these personal prerequisites for peak performance are often implicitly assumed rather than explicitly communicated.

**Table 1:** Overview of psychological theories relevant as framework models.

Theory	Short description
Self-Determination Theory (SDT) [12]	A theory of human motivation highlighting autonomy, competence, and relatedness as core psychological needs that promote intrinsic motivation and well-being.
Achievement Goal Theory (AGT) [13]	Focuses on the goals individuals pursue in achievement contexts: mastery (learning/improvement) vs. performance (demonstrating competence relative to others).
Expectancy-Value Theory (EVT) [14]	Performance, choices, and persistence are predicted by expectations of success and the subjective value placed on the task.
Grit (Perseverance and Passion for Long-Term Goals) [15]	Grit is the tendency to sustain interest and effort toward long-term goals despite setbacks.
Deliberate Practice Framework [16]	Expertise results from extended, structured practice focused on improvement, feedback, and pushing beyond current limits.
Social Cognitive Theory / Self-Efficacy [17]	Behavior and achievement are guided by self-efficacy beliefs — judgments of one’s capability to perform specific tasks — as well as by observational learning and reciprocal determinism.
Job Demands-Resources Model (JD-R) [18]	Performance and well-being result from the balance between job demands (workload and stressors) and job resources (support, autonomy, and feedback).
Conservation of Resources (COR) Theory [19]	Individuals strive to obtain, retain, and protect resources (time, energy, and social capital) and experience stress when those resources are threatened or lost.
Flow Theory [20]	Optimal experience arises when challenge matches skill, producing deep focus, intrinsic enjoyment, and high productivity.
Expectancy Theory (Vroom) — Work Motivation [21]	Motivation is a function of expectancy (effort → performance), instrumentality (performance → outcome), and valence (value of outcome).
Self-Regulation / Control Theories [22]	Successful goal pursuit depends on processes of planning, monitoring, feedback, and adjustment (meta-cognition, time management, and emotion regulation).
Identity and Communities of Practice [23]	Professional identity (e.g., “researcher”) forms through participation in communities of practice; identity shapes motivation, goals, and persistence.
Attribution Theory [24]	How people explain successes/failures (attributions: ability, effort, task difficulty, luck) shapes future motivation and behavior.

### 1.2 Academic performance: Dropout in academic career paths

Research on career path development has repeatedly shown high attrition rates among scientists pursuing PhD and postdoctoral careers [25]. Although no uniform prevalence data are available, dropout rates can be high, and career paths can be long. About 34% of PhD students in Europe don’t complete their studies within 6 years [26]. At Australian, British, Canadian, and US universities, the numbers range from 30% to 50% across disciplines [27–29]. Postdoctoral research, a key career bottleneck due to limited tenure-track roles, also shows high dropout rates [30,31].

### 1.3 Psychological factors and academic performance

Previous research suggests that several psychological factors can influence academic performance. Studies, for example, have examined interindividual differences between early-career scientists who plan to leave academia and those who plan to continue to pursue their academic goals [32–37]. This has revealed several personal and systemic factors. Key factors associated with completing a PhD, as reported in the studies, included high intrinsic motivation, positive relationships with supervisors, and social support. Among postdoctoral researchers, performance factors such as clear career goals and scientific publications appeared particularly important. Recent studies suggest that publications in high-ranking journals, content that focuses deeply on dissertation topics rather than pursuing novel research directions, strong networking (e.g., abroad), clear career goals, and mentoring are key to successfully continuing an academic career beyond the PhD [31]. Moreover, studies have found that individuals who finish their career choose career paths that match their skills, have high self-confidence, are stress-tolerant and compliant, and report higher life satisfaction [37].

Studies among doctoral candidates, postdocs, and supervisors reveal high performance expectations across all career stages. However, the views and expectations of doctoral candidates, postdocs, and supervisors can diverge. Many studies have found that early-career candidates regard responsibility for their own research as the primary driver of their decision to pursue doctoral or postdoctoral positions; they expect supervision and feedback and see the main challenge and concern as the time pressure of the work, especially the pressure to publish [38–41]. When researchers are asked in their roles as supervisors and mentors, specific expectations for early-career researchers become evident: studies have found that supervisors expect their teams to demonstrate technical and methodological competence, present research results in a scientific manner, and reflect critically on their research and performance [42].

Among these expectations, high self-efficacy—an individual’s belief in their ability to succeed—has been found to significantly enhance individual and team performance [43–45]. Theoretically, self-efficacy has been suggested to enhance performance by empowering individuals to manage challenges, seek innovative solutions, and avoid cognitive biases in decision-making [17,46].

Moreover, emotions, particularly achievement emotions such as joy, pride, fear, and shame, have been discussed theoretically as playing a key role [47]. For instance, joy can enhance motivation and persistence, whereas fear may inhibit performance if not properly managed. Theoretically, effective emotion regulation and a positive affective state are considered important factors in optimizing performance and motivation [47]. Individuals who can use their emotions constructively might be more resilient, which may help them achieve better performance outcomes. Recent research suggests that resilience and mental strength may be particularly helpful in boosting academic performance under pressure [48].

Despite many psychological factors positively impacting performance and well-being, recent survey studies report that young adults in academia experience daily stressors stemming from the pressure to continuously excel academically and professionally [49–51]. These challenges can intensify emotional burdens and an overwhelming fear of failure. Fear, stressors, worries, and health burdens do not simply disappear upon graduation but persist among PHD and postdocs. They might be perceived and accumulate differently between genders (women and men) or among international students at these career stages [49,52–54].

### 1.4 Gender and science

Many female researchers have been found to leave their careers early [55]. Of the remaining women, less than 30% hold leadership roles [56,57]. Gender-related differences can discourage young women from pursuing academic careers, especially after completing their PhD [56,58], although this may vary by scientific discipline and domain [59] or by cultural factors. In particular, the challenges of acculturation, seeking integration, and obtaining residence permits often place high stress and mental load on international students in the early years of their careers [60]. In addition, working conditions in science are challenging and do not always match private, still gender-related obligations. Gender biases in science are not independent from socially and culturally constructed gender stereotypes that arise early in the educational context [61,62].

## 2. Aim and scope of the present research

The aim of the present study is to explore the role of the psychological factors outlined in the previous sections in the early-career trajectories of PhD and postdoctoral researchers. In particular, the survey aims to explore how the PhD and postdoctoral researchers themselves judge these factors and evaluate their roles in achieving performance goals, satisfaction, mental health, and well-being. Thus, this survey places particular emphasis on participants’ expectations, i.e., how they perceive their performance and their skills, and competencies in achieving their qualification goals; the reasons they identify as barriers to achieving those goals; and what, in their view, might make one stay in or leave academia. The survey approach combines performance- and health-related psychological factors identified in the literature as relevant to academic performance (see Table 1 and the sections above). These factors have been considered important both theoretically and empirically, but have so far rarely been examined together. Doing so could provide insights into developing early-career training programs that integrate performance- and health-related factors to promote healthy academic peak performance.

Given that psychological factors might vary within and between PhD and postdoctoral researchers across a) time (starting a PhD or postdoc career or being about to finish it), b) the domain (study subject, discipline), and c) with sociodemographic factors (culture, language), and d) interact with gender, the present survey aimed to explore a homogeneous population with respect to language and educational system and time of career path while taking both PhD and postdoctoral career paths into account. Doing so limits the generalizability of the results and prevents transfer to broader, less homogeneous samples, while providing insights into the particular needs and views of PhD and postdoctoral students with similar preconditions. Nevertheless, the results of this survey assessment will help identify how particular factors support or hinder PhD and postdoctoral researchers, both women and men, with similar social preconditions in pursuing and achieving their performance goals across scientific domains and disciplines, including STEM and STEAM (science, technology, engineering, mathematics, and arts) focusing particularly on Germany, Austria, or Switzerland.

In summary, the following research questions are explored:

a Investigate participants’ performance expectations, beliefs, attitudes, motivation, and emotions, especially self-efficacy, performance motivation, and achievement emotions, perceived stress and workload, and well-being in achieving academic goals.
b Investigate the participants’ satisfaction and meaningfulness with their own work, effort, and skills; their motivation and engagement in their academic careers.
c Explore the participants’ satisfaction with supervision and experience with early career tools such as mentoring or academic coaching.
d Investigate the impact of gender on academic performance expectations and academic career paths.
e Investigate if expectations and evaluations differ between PhD or postdoctoral career paths and between participants planning at the time of the survey to leave or stay in academia after reaching their career goal.

## 3. Methods

### 3.1 Participants and procedure

From September to December 2025, academic volunteers participated anonymously in an online survey, labeled MERIT – empowering academia, and provided written informed consent. N = 489 registered for the study, and N= = 475 filled in the survey. Of these, N = 298 fully completed the questionnaire. Some participants completed the survey but did not answer all items, using the answer option (prefer not to answer) or leaving one or more items blank (as allowed in the survey). Table 2 provides an overview of the sample sizes.

**Table 2:** Survey participants, responses provided, including gender preferences.

Participant sample	Responses provided, “prefer not to say,” or no answer
**Participants who filled in the survey**	**N** = **475 (n** = **164 women, n** = **287 men, n** = **4 other, N** = **20 (preference: not to say or no answer)**

The online survey was conducted with a focus on early-career and postdoctoral researchers. As outlined in section 2, to ensure comparability across academic career stages, the survey was administered to participants in three countries with similar educational systems and the same language: Germany, Austria, and Switzerland. A master’s or bachelor’s degree, or an equivalent from countries worldwide, was not an exclusion criterion.

The professional platform Prolific (https://www.prolific.com/) was used to recruit participants in accordance with the privacy policy and the standards of European data protection law (GDPR). Prolific allows prescreening of participants based on sociodemographic variables such as language, country, age, or education. Therefore, the country prescreen was set to reach volunteers from Germany, Austria, and Switzerland. Prescreening on Prolific would also have allowed gender-based selection to achieve an equal gender distribution. This filter was not set because it might artificially obscure gender differences by creating the impression of gender equality in participation.

At the beginning of the survey, participants were thoroughly debriefed about the study. They were provided with detailed written information about the procedure and how their data would be treated and stored. In the first survey phase reported here, the survey targeted academic individuals in the qualification phase who had started a doctorate or postdoctoral activity and had not yet completed it. Inclusion criteria were age 18 or older and fluency in German, spoken as a native language. The decision to offer the survey in German was driven by a focus on the target countries, not by excluding international students or students with proficiency in German and meeting the eligibility prerequisites discussed above. Participation was voluntary and anonymous; participants provided written informed consent.

#### Ethics statements.

The anonymous survey was conducted in accordance with ethical recommendations and guidelines. Inclusion criteria were age 18 or older and fluency in the target language. Additionally, participants were recruited anonymously; i.e., no personal information (IDs, names, emails, etc.) that could risk deanonymization was collected or stored in the data poll. Moreover, participants could choose to leave items unanswered or tick the “prefer not to say” option if they preferred not to answer (see details above and Table 2). The participants received detailed instructions regarding the survey’s purpose and provided written informed consent; they were financially reimbursed for their participation. All participants voluntarily participated and provided written informed consent prior to completing the survey. At the beginning of the survey, they were given a detailed debriefing on how their data would be handled and stored in line with GDPR. They were informed that they could withdraw from the study at any time during the survey without providing reasons or incurring any confidentiality-related consequences. All registered participants had equal access to the survey and received the same financial reimbursement for participation.

### 3.2 Procedure survey

In total, the survey comprised yes/no items and items requiring evaluation on VAS-like Likert scales (1–5, 1–9, or 1–10) or percentage scales (0–100%). Additional items were open-ended questions that participants could answer with a few keywords or a few sentences. Sociodemographic data included questions about gender (woman, man, other, or “prefer not to say”), current academic position, country, and academic discipline, years of PhD or postdoctoral qualification, and prior degrees and academic background.

As shown in Table 3, the survey items captured the key psychological factors discussed in the previous sections as main performance modulators, including candidates’ performance expectations and satisfaction, self-efficacy, self-control, achievement emotions, and performance motivation. Moreover, it included questions about the regularity and satisfaction with supervision, attitudes toward careers, perceived career barriers, coping with stress and emotion as resilience factors, and experiences with coaching as an early-career intervention.

**Table 3:** Psychological factors grouped into thematic domains, constructs, and contextual variables as assessed in the MERIT survey. The standardized survey was administered to early-career researchers to examine the role of the individual performance factors summarized in Table 3, including performance expectations, beliefs in competencies, mental health, resilience, self-efficacy, and supervisory support. For details, see Sections 2, Methods, and Results.

Psychological Factors Construct /Concept Thematic Domain	Conceptual Definition (Assessed via both closed and open-ended items, see text for details)
Performance Expectations	Anticipated levels of personal performance and perceived likelihood of achieving qualification and project-related goals.
Self-Efficacy	Beliefs about one’s capability and skills to achieve one’s performance and academic goals.
Achievement Emotions	Emotions associated with learning, performance, and goal pursuit.
Motivation	Intrinsic versus extrinsic motivational orientations underlying engagement in qualification activities.
Satisfaction	Perceived satisfaction with one’s academic achievement and effort.
Meaningfulness of Work	The extent to which individuals perceive their work as purposeful, valuable, and aligned with personal significance (assessed via open-ended questions).
Mental Strength	Evaluation of having the psychological resilience, cognitive focus, and perseverance in the face of pressure or adversity to achieve one’s academic goals.
Self-Regulation	Capacity to manage thoughts, emotions, and behaviors in alignment with long-term goals.
Knowledge, Skills, and Competencies	Self-assessment of the extent to which individuals can work independently and apply their expertise to achieve qualification and project goals.
Gender and Role Expectations	Perceptions of gendered norms, stereotypes, and role expectations that may shape career trajectories (open-ended questions only).
Satisfaction with Supervision	Evaluation of the adequacy, quality, and responsiveness of supervisory support (both closed and open-ended items).
Career Attitudes	Beliefs and orientations toward future career pathways, opportunities, and aspirations (both closed and open-ended items).
Career and Work Barriers	Experiences with structural, interpersonal, or personal obstacles encountered in earlier career stages (both closed and open-ended items).
Coping with Stress and Emotion	Strategies and competencies for managing stress, emotional distress, and challenging situations (resilience factors), (open-ended items).
Experiences with early career interventions/tools	Perceived relevance and experience with early career tools such as coaching, mentoring, or training (both closed and open-ended items).

### 3.3 Data analysis

Data were analyzed descriptively before advanced analysis. For each survey item, percentages, means or medians, standard deviations (SDs), and confidence intervals (CIs) are reported. The data were then examined for gender distributions, potential biases, and differences between genders and between PhDs and postdocs to explore challenges in career trajectories. Next, differences between participants planning to leave or stay in academia (groups “stay” and “leave”) were analyzed with respect to the psychological factors summarized in Table 3, including expectancies, satisfaction, motivation, self-efficacy, and achievement emotions.

Analyses addressing the research questions included parametric and nonparametric tests, as well as factorial designs with group as the between-subjects factor. Planned comparisons (where appropriate) were used to reduce false positives in multiple comparisons. Response patterns and relationships among answers in psychological domains (see Table 3) were identified via correlation analysis. Analyses included responses from participants who fully completed the questionnaire (N = 298) and, where available, from participants who completed the item, including answers indicated as ‘no answer’ or ‘prefer not to answer.’ Open-ended responses were analyzed semi-automatically using a combination of quantitative and qualitative methods. To this end, answers were coded into thematic categories and quantified (for details, see section 4.4). The quantitative analyses were conducted using the software Statistica (https://www.satistica.com/), XLSTAT (https://www.xlstat.com), or SPSS12 (IBM, https://www.ibm.com/de-de/products/spss-statistics). Qualitative analyses for open-ended questions were conducted with MAXQDA (https://www.maxqda.com).

## 4. Results

### 4.1 Descriptive statistics

#### 4.1.1 Gender and career paths.

Of N = 475 participants (responses), 63% were men, 36% were women, and 1% were other. Participants were from Germany (78%), Austria (9%), and Switzerland (4%), and 9% were from other countries. The participants reported pursuing academic goals at public universities (54%), research institutions (31%), or private universities or universities of applied sciences (15%). The majority of respondents reported pursuing their PhD/postdoc for 2–3 years, followed by 1 year, with a few reporting longer durations. Of the respondents, 53.6% held a PhD, 46.4% were postdocs (4% were habilitated and 10% already applied for professorships). About 57.3% reported a university position, 6.3% held research scholarships, and 36.4% reported being funded by their own research positions; see Table 4. Of the participants who reported their scientific domain, a wide range of domains was represented, including engineering, computer science, physics, management and technology, maths, neuroscience, biology, medicine, psychology, arts, and law, suggesting that STEM and non-STEM subjects were nearly equally represented (53% vs 47%).

**Table 4:** Sociodemographic/contextual variables and their distribution: number of answers (count) and in percent (%).

Category	Count (N)	Percentage (%)
Total Participants who filled in the survey	**475**	**100%**
Gender	**455**	**100%**
- Men	287	63%
- Women	164	36%
- Other	4	1%
- No Answer/ Prefer Not to Say	20	--
Country of Employment	**475**	**100%**
- Germany	370	78%
- Austria	43	9%
- Switzerland	19	4%
- Other	43	9%
Type of Academic Position	**475**	**100%**
- Public University (state)	259	54%
- Research Institution	146	31%
- Private University	32	7%
- University of Applied Sciences	38	8%
Academic Qualification Status	**411**	**100%**
- PhD Candidates (actively pursuing)	247	53.6%
- Postdocs (including those pursuing habilitation or a professorship)	116/48	46.4%
Funding Source for Academic Qualification	**475**	**100%**
- University Position	272	57.3%
- Scholarship	30	6.31%
- Own funding of research position	173	36.4%

#### 4.1.2 Satisfaction with one’s academic achievements.

When asked about their academic satisfaction (Likert scale: 1 = not satisfied, 9 = satisfied), respondents (N = 311) reported being satisfied (scores of 6–7; see Fig 1). A few respondents were either completely satisfied or completely unsatisfied. Regarding satisfaction with progress toward PhD or postdoc goals, PhD candidates reported greater confidence than postdocs (see Fig 1).

**Fig 1:**
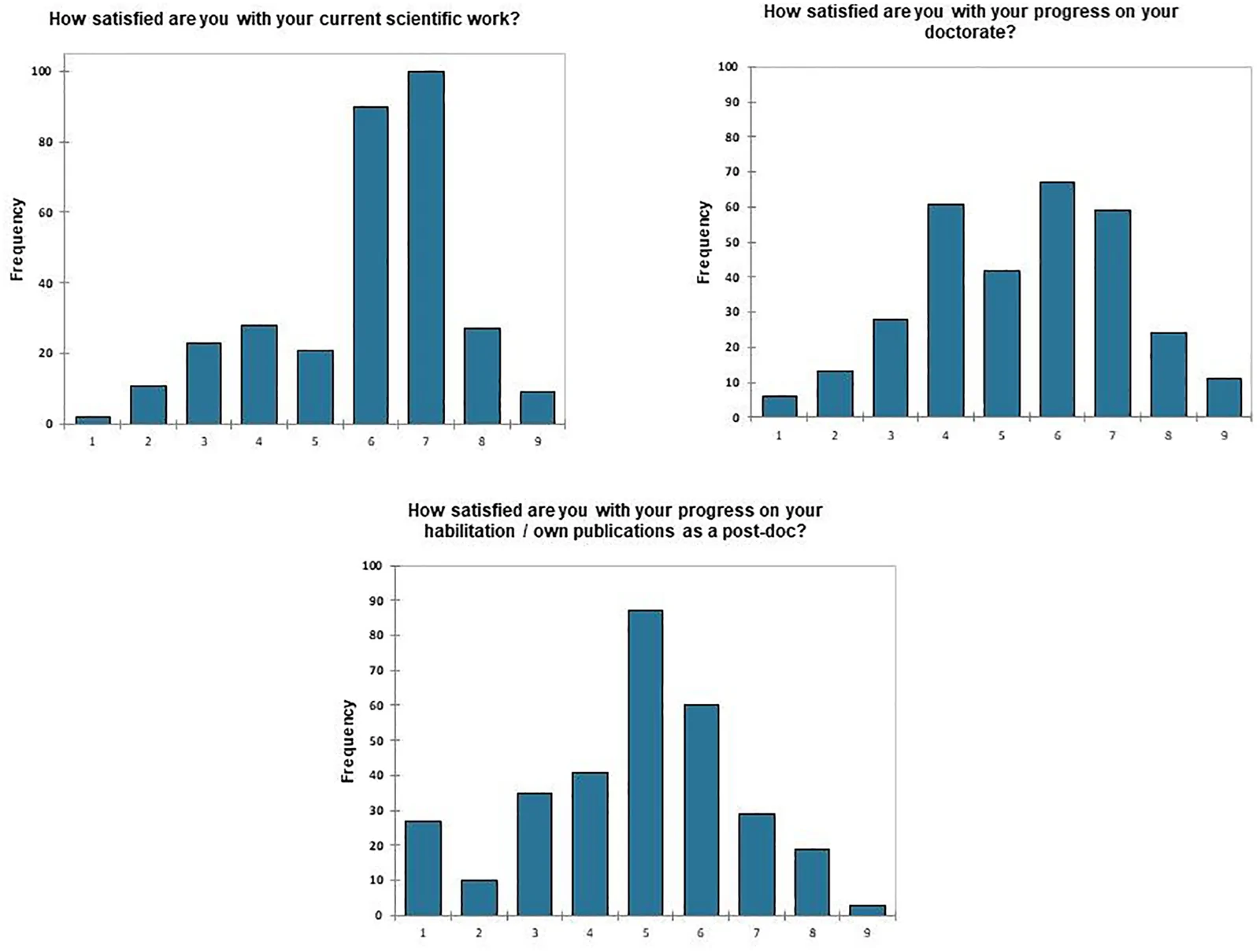
Overall satisfaction of respondents (N = 311) with their academic work, and their own qualification as PhD (doctorate) and postdoc. Scale from 1-9 (not satisfied – absolutely satisfied, satisfaction in percent).

#### 4.1.3 Beliefs about self-efficacy, self-control, motivation, and emotions as performance boosters or performance obstacles.

Participants could select up to 12 competencies, resources, and skills from three categories: academic skills; engagement and self-confidence; time management and stress; or social and academic support. Most agreed that their own efforts (60%), time (54%), own encouragement and optimism (49%), and methodological skills (33%) are key to academic success. Luck (89%), fewer social demands (50%), and supervisor encouragement (69%) were seen as least influential. Despite this, social interaction with colleagues and supervisors was rated 5–7 by 30% of respondents, indicating its importance. See Table 5.

**Table 5:** Evaluations of own competencies, resources, and skills, rank-ordered and percentage of agreement (%) among respondents; for details, see section 4.1.3.

Category	Factor	Percentage of Agreement	Rank
**Most Significant Factors (Position 1)**			
**Own Academic Competencies and Skills** **Time and Stress Management** **Own Engagement and Self-Confidence**	Greater Effort	60%	1
	More Time	54%	1
	Hope and Optimism in one’s achievement	49%	1
	Better Methodological Qualifications	33%	1
**Less Significant Factors (Position 12)**			
**External Factors** **Social and Academic Support and Supervision**	Luck	89%	12
	More Encouragement from Supervisors	69%	12
	Fewer Social Demands	50%	12
	More Interaction with Colleagues and Supervisors	30%	12

When asked about factors that support career achievement (0–100%), the answer option “already giving the best” achieved 71% (0–100%), “reaching goals through effort and doing even more to succeed” was rated 68%, and “support from others” was 63.5%. Participants reported spending an average 58% of their time on their own academic work and rated their academic goals as very important to them (see Table 6). These beliefs and expectations were also reflected in responses to questions about self-control, self-efficacy, performance motivation, the ability to motivate themselves toward their academic goals, their current experience and feelings of achievement, and the degree of frustration, fear, and worry during academic work. As shown in Table 6, self-control and intrinsic motivation for working alone received the highest scores, followed by experiencing both positive and negative emotions equally often, and feeling worried and fearful about completing academic goals and career. Participants experienced frustration about 62% of their work time (see Table 6).

**Table 6:** Evaluations of own effort, self-control, self-efficacy, achievement emotions, frustration, fear and worry, and performance motivation from the MERIT survey. Number of respondents (N), Mean, median, standard deviations (SD), and confidence intervals (CI, + /- 95%).

Questions	N	Mean	Median	SD	CI (+95%)	CI (−95%)
**Evaluation of Own Effort**						
How important are your academic goals to you? (1–9)	308	6.70	7.00	1.82	1.69	1.98
How much time do you spend daily on your academic work? (0–100%)	308	58.06	62.00	20.94	19.40	22.74
How much are you already making an effort to achieve your qualification goal? (0–100%)	311	70.98	75.00	18.89	17.51	20.50
Do you think you can do even more to reach your qualification goal? (0–100%)	311	68.22	72.00	23.92	22.17	25.96
**Self-Efficacy and Self-Control**						
How strongly do you believe that you have the skills and control over your qualification goals? (1–9)	308	6.02	6.00	1.67	1.54	1.81
To what extent do you believe that you can achieve your qualification goal solely through social and emotional support from others? (0–100%)	311	51.68	55.00	24.62	22.82	26.72
Do you think others could support you better in achieving your qualification goal? (0–100%)	311	63.46	65.00	22.26	20.64	24.16
Do you work autonomously in your academic work? (1–9)	308	6.40	7.00	1.83	1.69	1.98
Are you highly dependent on the external results of others in your academic work? (1–9)	308	4.60	5.00	1.92	1.78	2.09
Do you prefer to work alone?	308	6.17	6.00	1.96	1.81	2.12
Do you prefer to work in teams?	308	5.03	5.00	1.97	1.83	2.14
**Worry & Fear**						
How worried or anxious are you about your academic career? (1–9)	308	5.89	6.00	2.16	2.01	2.35
**Frustration**						
How often do you experience frustration? (0-100%)	308	61.72	63.00	22.85	21.17	24.81
**Achievement Emotions**						
How strongly do you experience positive emotions regarding your academic achievements? (1–9)	308	5.67	6.00	1.85	1.72	2.01
How strongly do you experience negative emotions regarding your academic achievements?	308	5.64	6.00	1.92	1.78	2.08
						
**Motivation**						
How would you rate your motivation for your academic work? (1–9)	308	6.19	6.00	1.82	1.69	1.98
Do you need a lot of encouragement from others to work on your academic tasks? (1–9)	308	4.65	4.50	2.04	1.89	2.21
Do you need many positive successes to continue pursuing your academic work? (1–9)	308	5.62	6.00	1.75	1.62	1.90
Can you motivate yourself to work on your academic tasks independently? (1–9)	308	6.21	6.00	1.76	1.63	1.91
How responsible do you feel for achieving the goals in research projects you are involved in? (0–100%)	308	68.05	70.00	20.16	18.69	21.89
						

#### 4.1.4 Expectations about academic supervision.

While 70% of participants who answered the question (N = 311) reported receiving regular supervision, 30% did not feel they were regularly supervised, and only 2.4% of respondents (all PhDs) reported receiving regular supervision to a high degree (Likert scale: 1–9). Only 12% felt everything was fine, with 4% preferring less content supervision and 53% wanting more. About 52% sought additional personal feedback. 52% wanted more empathetic supervision. Just 26% believed supervisors needed more institutional support; 34% thought extra initiatives were needed. In response to an open-ended question, only 11% of respondents had attended courses such as time management or stress management, and 2% had started or completed early-career programs (see Figs 2, Fig 3 and 4).

**Fig 2:**
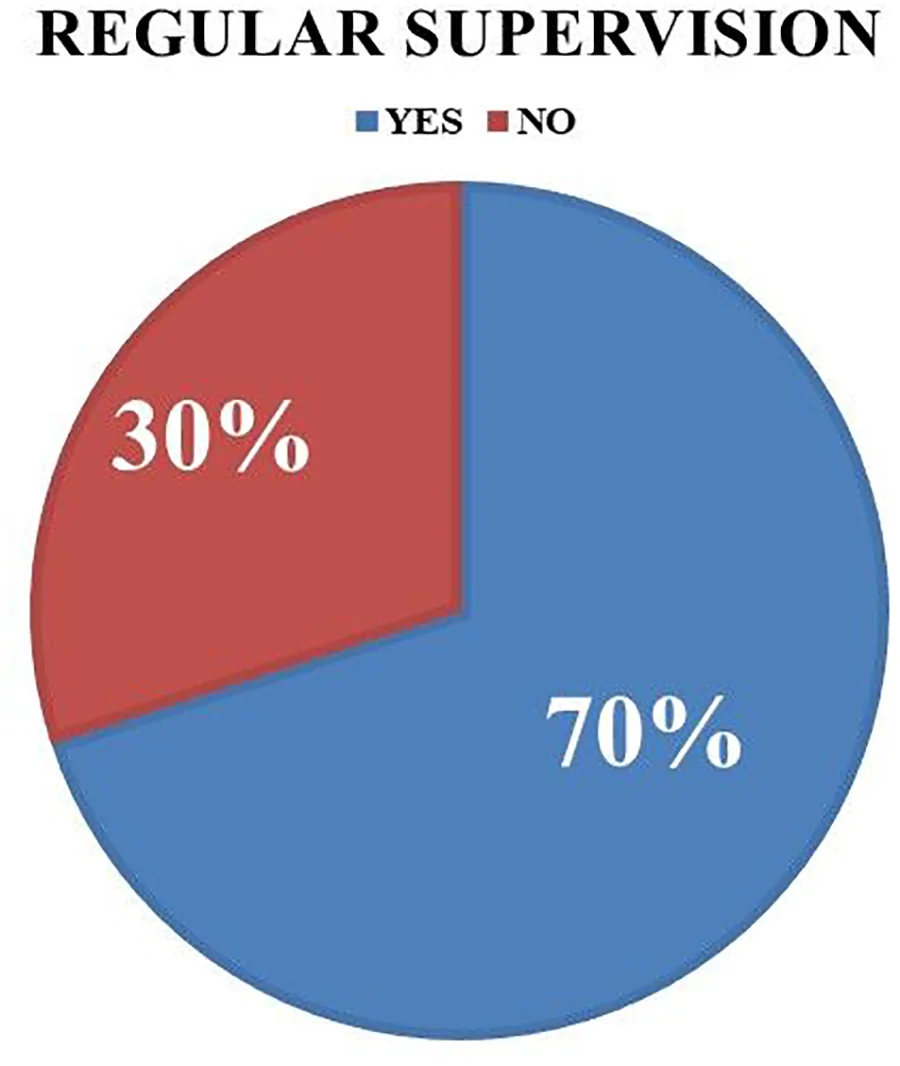
Percentage of answers of the participants (N = 311) regarding the question of whether they are receiving regular supervision for their academic career (PhD/postdoc).

**Fig 3:**
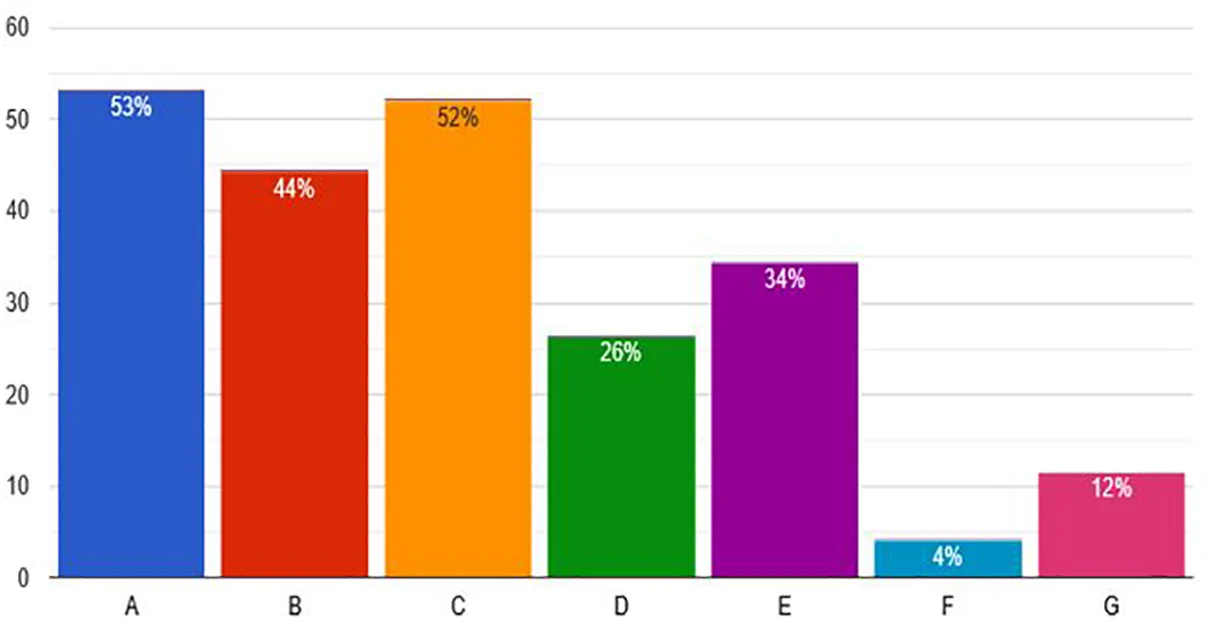
Satisfaction with supervision. A: More time for scientific discussions; B: More empathy; C: More personal content-related promotion and support; D: More recognition of supervision by third parties; E: Promotion of supervision programs to strengthen supervision; F: Less content-related supervision; G: Everything is fine. x-achses (answers in percent (%)). N = 311 respondents.

**Fig 4:**
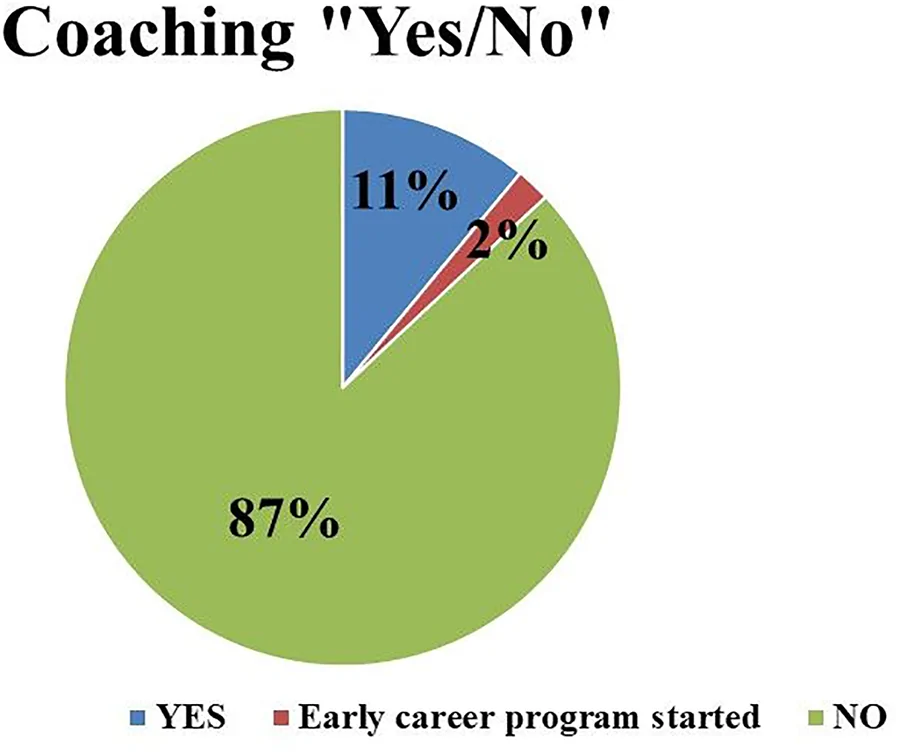
Percentage of the participants (N = 292) who reported early career or coaching experience as a PhD or postdoc.

#### 4.1.5 Academic goals – expectancies regarding present and future.

Participants’ confidence in reaching their academic goal (PhD or postdoc) had a median of 6 on a 1–9 scale (mean: 6.2; SD: 2.1; range: 1–9). When asked about future career goals, overall, 19% of PhDs and 10% of postdocs who answered the question (N = 326) planned to stay in academia; whereas 19% of the PhDs and 6% of postdocs) planned to stay in academia. Some participants (N = 209), in response to the open-ended questions, explained why they might leave academia after completing their academic qualifications and shared their views on the main daily obstacles hindering their academic goals. About half of the participants answered these questions (N = 255). Their responses were analyzed using MAXQDA, coded into themes: a) lack of career prospects, b) poor pay, financial issues, contract limits, c) lack of motivation, boredom, or dissatisfaction, d) lack of support, e) non-academic obligations, f) ambitions outside academia, g) poor work-life balance, h) mismatch between expectations and reality. Common issues included time constraints, financial and emotional challenges, work and private responsibilities, communication problems (including supervision) (see Tables 7 and 8).

**Table 7:** Summary: Reasons provided by the participants, who provided answers (N = 209) that could make one leave academia after the PhD or postdoctoral career, sorted according to their frequency of occurrence. The most frequently reported reasons are listed first, followed by those with the lowest frequency.

Theme	Absolute Frequency Ranking
**Job, Employment, and Financial Conditions**	Position 1
- Job insecurity, low payment, and employment situations, e.g., uncertainty about staying in academia	
- Limited job opportunities in academia and better prospects in the private sector	
- Financial disadvantages, economic burdens, and a need for better payment	
**Personal Preferences**	Position 2
- Desire to work in the industry or the private sector	
- Preference for practical work and clinical practice	
**Work-Life Balance**	Position 3
- Poor work-life balance and demanding working hours	
**Motivation and Engagement**	Position 4
- Lack of passion for research and academic work	
- Burnout and high expectations	
**Administrative and Bureaucratic Challenges**	Position 4
- High bureaucratic burden, administrative tasks	
**Career Development Concerns**	Position 5
- Limited opportunities for advancement, supervision	
- Challenges in transitioning to industry	
**Social and Cultural Factors**	Position 6
- Issues faced as an international researcher	
- Hierarchical structures and lack of development	
**General Challenges**	Position 7
- General dissatisfaction with academic conditions	
- Stress from competition and job insecurity	

**Table 8:** Summary: Reasons provided by the participants, who provided answers (N = 255), for the most significant continuous barriers and challenges experienced that hinder them from achieving their daily academic goals. The most frequently reported barriers are listed first, followed by those with the lowest frequency.

Theme – Barriers	Absolute Frequency in descending order
**Time Constraints**	Position 1
**Private Responsibilities**	Position 2
**Work-Related Stress and Financial Worries**	Position 3
**Motivation Issues**	Position 4
**General Distractions**	Position 5
**Administrative Tasks**	Position 6
**Dependence on Others**	Position 7
**Emotional State**	Position 8
**Supervision Issues**	Position 9
**Bureaucratic Challenges**	Position 10
**Career Development Concerns**	Position 11
**Social and Cultural Factors**	Position 12
**General Challenges**	Position 13

### 4.2 Differences between women and men pursuing a PhD or Postdoc career

Statistical comparisons between participants reporting binary gender (woman vs. man; women = 112, men = 118) showed no differences across survey items or psychological factors depicted in Tables 2, 5, and 6. T-tests and Mann-Whitney U tests revealed no significant differences in academic goal satisfaction, motivation, effort, self-control, self-efficacy, or emotions between males and females. Trends toward significance were seen in worry, fear of not completing the qualification, and frustration. Women reported overall more worry and anxiety (t = 4.83, df = 298, p < .05) and more frustration than men (t = 1.85, df = 298, p = .06).

When considering career stage, concerns did not differ between PhDs and postdocs; women reported more worries than men at all stages. The effects were significant in both groups (PhD: n_women_ = 66, n_men_ = 95, t = 3.86, df = 159, p = .0002; Postdocs: n_women_ = 46; n_men_ = 93, t = 2.93, df = 137, p = .003). Women pursuing a PhD believed they could put in more effort than men (t = 2.23, df = 161, p = .003). Postdoc women showed a trend to report fewer positive and more negative emotions than men (n_women_ = 46; n_men_ = 93: both positive and negative emotions: t = −1.8 or – 1.8, df = 137, p = .07). Women also worked alone more often during the postdoc stage (t = 2.15, df = 169, p = .003). They reported needing more positive feedback than men during the PhD (t = 2.46, df = 169, p = .015), but during the postdoc stage, men valued continuous positive feedback and rewards more for career advancement than women or male PhDs did.

Women reported planning to leave academia more often than men. After their PhD, 20.9% of women and 11.22% of men wanted to leave, while 41.67% of women and 18.52% of men wanted to leave after their postdoc. More women (23.9%) than men (17.34%) reported wanting to stay during their PhD, and similarly, 25% of women versus 14.3% of men during the postdoc stage. Uncertainty about staying or leaving was evident: 55.22% of women and 71.42% of men were uncertain during the PhD, and 58.33% of women and 67.20% of men during the postdoc. That is, 55.22% of women and 71.42% of men had not decided at the time of the survey whether they plan to stay in or leave academia after completing their PhD or postdoc.

When looking at gender parity in single domains such as computer science, engineering, psychology, medicine, or biology, the following gender distribution ratios (%) were observed: Computer Science: 81:19 (men vs. women), Engineering: 90:10 (men vs. women), Neuroscience: 33:67 (men vs. women), Psychology: 51:47 (men vs. women, other: 2%), Medicine: 15:85 (men vs. women), Biology 51:49 (men vs. women), Education: 46:54 (men vs. women), Law & Management: 50:50 (men vs. women).

### 4.3 Differences between PhDs and Postdocs

Comparisons between PhDs and postdocs revealed only a few significant differences. Significant differences were found in the self-reported confidence in one’s academic achievement and in beliefs about the skills required to achieve academic goals and master the work environment. Postdocs were less confident in meeting the qualification requirements than PhDs, t = −2.89, df = 309, (n_Postdocs=_144, n_PhD_ = 167), p = .004. However, they were more satisfied with their achievements than PhD candidates, t (309) = 1.99, p = .046. In addition, postdocs believed less than PhDs that encouragement could help them attain their academic goals, t = −2.75, df = 311, (n_Postdocs=_146; n_PhD_ = 167), p = .006; they felt to work significantly less self-determined than PhD candidates, t = −2.36, df = 306, (n_Postdocs=_143, n_PhD_ = 165), p = .018.

### 4.4 Differences between groups who plan to stay or leave academia, or are uncertain

The following comparisons were made across three groups who answered the yes/no questions about decisions to leave or stay. The groups comprised PhD or postdocs who were planning to leave academia after completing their PhD or postdoc (group “leave”), versus those who planned staying (group “stay”), and those who had not yet decided (group “insecure”) and therefore could not answer this question, comprising N = 50 (16.27%) postdocs, and N = 106 (34.48%) PhDs. Comparisons between these three groups yielded the following significant differences. Participants who reported that they would like to stay were significantly more satisfied with their academic work than those who decided to leave (t = 2.60, df = 151, (n_stay=_84, n_leave_ = 69), p = .01). To them, their academic goals were also more important than those who planned to leave (t = 4.34 df = 150, (n_stay=_83, n_leave_ = 69), p = .0002). They were more motivated than those who wanted to leave (t = 2.31, df = 150, (n_stay=_83; n_leave_ = 69), p = .022) and felt overall more responsible for the academic project work (t = 3.72, df = 150, (n_stay=_83; n_leave_ = 69), p = .0002).

Significant differences were observed across the three groups in confidence in qualifications, satisfaction with academic work, and motivation to pursue goals. Kruskal-Wallis ANOVA showed that the group planning to stay prioritized academic goals most H(2, 308)=18, p < .0001, was most satisfied with their academic achievements H(2, 311)=5.6, p = .05, and was most confident in self-motivation H(2, 308)=5.77, p = .056, whereas the insecure/uncertain group valued work exchange within the work group as beneficial for achieving academic success, H(2, 308)=5.69, p < .01. The group “stay” also felt responsible for the overall project goals (rather than just focusing on their own objectives) compared to both other groups, H(2, 308)=15.91, p = .004.

However, the three groups did not differ significantly in their judgments and evaluations of the other factors. They also did not differ in their evaluations about how much effort they had already put into achieving their goals, nor in whether they could still put more effort and strength into them. However, as described above, significant differences were found among the three groups in their evaluations of the personal importance of academic goals. Chi-square tests showed no significant differences between PhD and postdoc candidates (χ² = 0.3, df = 1, p = .5) or between women, men, and those not reporting their binary gender (χ² = 8.2, df = 2, p = .2) in their distributions across the groups.

### 4.5 Exploratory analysis: Relationship between psychological variables

To explore potential relationships among the psychological factors, correlations (statistically tested using Kendall’s tau or Spearman’s rho) were computed, revealing significant relations in self-reported effort, beliefs, and expectations. Participants’ beliefs about achieving goals through effort and support correlated significantly (p < .001): greater self-efficacy was associated with openness to external support and willingness to work harder. Positive emotions correlated with most psychological domains, including self-confidence, progress towards reaching PhD/postdoc goals, performance motivation, and belief in success. It was also related to negative emotions, but not to worries about performance, which themselves correlated with negative feelings (p < .01). Most findings remained significant after multiple-comparison correction (see Figure 5).

**Fig 5:**
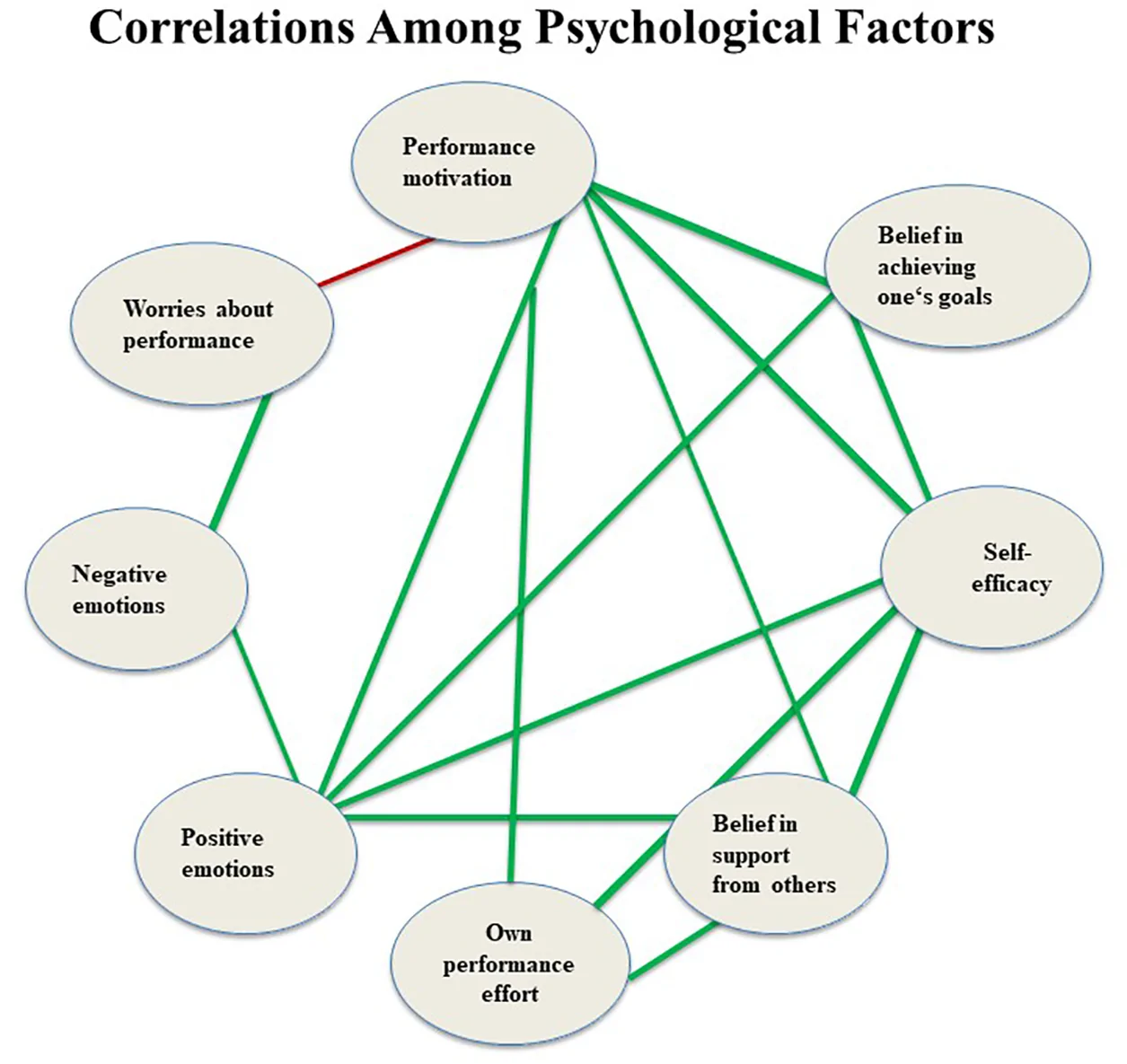
Correlations between psychological factors. Legend: Green lines: positive correlations p < .01, red lines: negative correlations p < .01.

## 5. Discussion

This survey explored young career researchers’ performance expectations and their views on which psychological factors they consider relevant to achieving their academic performance and goals when pursuing a PhD or postdoctoral career path in countries with similar language, educational systems, preconditions, and precarities regarding PhD and postdoctoral career trajectories [63]. As highlighted in the OECD Reports 2023 [63–65], there are ongoing trends and concerns about increasing attrition, particularly at the postdoc stage (e.g., prolonged postdoc periods), with rising numbers of PhD candidates but overall limited permanent academic positions. In summary, these conditions can foster negative expectancies among young career researchers due to career uncertainty, delayed career stability, a competitive early-career track, and credibility gaps. Expectancies among young career researchers might be state-dependent, begin early in their careers, and change over time. Therefore, the present survey and its findings can provide significant insights into PhD and postdoc researchers’ expectations for their academic goals, views on how best to achieve them, and worries and concerns. By focusing the survey on psychological factors discussed in the literature as modulators of academic performance yet seldom investigated in conjunction, the present survey provides a first evaluation of how early-career researchers (PhD students and postdocs), having begun and consolidated their career paths, assess these factors.

### 5.1 Expectations concerning academic performance and goals

Overall, survey participants were satisfied with their academic work and their achievements. Few were completely satisfied or completely dissatisfied, as shown in Fig 1. This pattern was observed across evaluations of the performance factors (Tables 4–6). The results suggest that participants who were pursuing their PhD and postdoc careers at the time of the survey (the majority of PhDs or postdocs reported 2–3 years of experience, followed by a year of experience) feel confident in their academic competencies and strategies for achieving their academic goals. When asked to rank factors by importance, the ranking clearly reflected the candidates’ belief that their own competencies, effort, motivation, and emotions, such as optimism and courage, are predictors of performance in achieving their academic goals. However, as a group, postdocs were overall less confident in their qualifications than PhD candidates, though they were more satisfied with their achievements than PhD candidates. The analysis of responses from candidates planning to leave or stay in academia revealed that participants’ intentions to leave or stay distinguished them from one another and from those who, at the time of the survey, were still uncertain about whether to stay or leave after reaching their career goals. The group that planned to stay showed higher work satisfaction and greater confidence in their abilities, driven by personal control, effort, and skills, compared to the group that intended to leave or were uncertain. PhD and postdoc candidates who had not yet decided whether to stay or leave reported more often than the other two groups that team support could enhance their academic success. While all groups valued their own effort, the group that intended to stay in academia rated academic goals as more important and felt a greater sense of responsibility for the project’s goals beyond their own. No differences emerged between PhD and postdoc candidates, suggesting that decisions about staying in or leaving academia, or having no clear view of it so far, can arise at any career stage. Moreover, regardless of career stage (PhD or postdoc), more women (percentage scores) planned to leave academia than men did, and 55.22% of women but 71.42% of men had not yet decided whether to remain in or leave academia after finishing their PhD or postdoc. This supports previous findings that women leave academia early [55,61]. However, Chi-Square tests did not reveal a significant gender difference.

The observation significantly adds to the discussion of the role that intentions in career planning might play in the expectations of both female and male participants regarding their own achievements, barriers, motivation, emotions, and well-being. Most reports (e.g., in Germany) distinguish among three PhD or postdoc stages (start, consolidation, and finishing periods) and suggest that these periods are critical for predicting attrition and delays in time-to-degree [66–68]. Expectations and self-assessments of early-career researchers may vary over time. Thus, the intentions reported by the survey participants may reflect only a snapshot and may change over time, as the survey study is silent on intradividually longitudinal changes and the long-term impact of the observed differences. The results, therefore, support the suggestion that the intentions of young career researchers should be followed longitudinally in future studies. This could help determine whether the more positive group regarding decisions to stay actually stays or may become dissatisfied with their intended career progression at a later stage, and whether the undecided yet uncertain early-career researchers might change their expectations as they become more certain about whether to stay or leave academia. The present results suggest that career path decisions occur both within the PhD and postdoc trajectories and are therefore relevant to monitor throughout the entire career path.

### 5.2 Gender as push and pull factor

The gender distribution, with only 36% of participants being women, warrants attention. The survey was delivered via an online research platform that allows prescreening of participants based on sociodemographic variables such as language, country, age, or education. Prescreening would also have allowed gender-based selection to achieve an equal gender distribution. This prescreening was not set, as it might artificially create the impression of gender equality in participation. Moreover, when examining gender distributions by the scientific domain in which participants reported pursuing their career paths, either more female or more male participants were represented in the survey. For example, in psychology and biology, more female than male participants registered for the survey, whereas in engineering and computer science, more male than female participants registered; in law and politics, women and men were equally represented (see section 4.2). Taken together, these distributions align with the gender distributions reported in the literature for the STEM and STEAM domains [69]. Nevertheless, overall, more men than women took part in the survey. These differences in gender distributions in the survey would not have been observed if artificially generated gender prescreeners had been used. The overall distribution is lower than that reported in statistical reports on gender in early-career paths in the three target countries, which report nearly gender parity at the PhD level (outside STEM disciplines) and a significant drop in the women-to-men ratio at the postdoc level, especially in STEM disciplines [69]. Whether the gender ratio in the present survey may point to an issue reported in previous research, suggesting that women may be less encouraged to participate in surveys on career decisions [70], despite the current survey being equally accessible to male and female participants, requires further research.

Interestingly, in the present survey, women who reported a binary gender did not differ significantly from men on many performance factors. Statistical comparisons showed no significant gender-related differences in performance motivation, effort, self-control, or self-efficacy. However, significant gender differences in self-reported emotions were found, with women reporting greater worry and anxiety about not completing their qualification. Moreover, and in line with previous studies [71], women reported experiencing frustration more often than men. Comparing gender effects across career paths showed that, as postdocs, women reported fewer positive and more negative emotions than men. They worked in a self-determined way, whereas men valued continuous positive rewards more than women and more than they did during their PhD.

This suggests interesting changes across the career path in the beliefs and attributions about what women and men believe they need to support their careers. As explored in correlation analyses, achievement emotions were correlated with many performance factors (see Fig 5 for an overview). Thus, the results can inform early career initiatives to be particularly alert to the role of achievement emotions, their experience among women and men across career paths, and how to promote positive emotions and avoid negative emotions, worry, and frustration in the service of reaching peak academic performance and well-being in a gender-sensitive, nondiscriminative manner. The observation that at the postdoc stage, women in the present survey reported taking responsibility for their careers and working more self-determinedly, while men as postdocs showed more reward-oriented behavior, suggests differences between genders in seeking and receiving feedback as PhD and postdoc [72]. This should be explored further because studies have found that women often receive less scientific feedback than their male colleagues, a pattern that may be driven by gender stereotypes [73,74]. Therefore, coaching tools and short-term psychological interventions, such as those summarized in Table 9 [see also 75], could serve as treatment options.

**Table 9:** Conceptual research approach (MERIT) to empower and enhance scientific performance and health in higher education via standardized assessment and interventions (see also [75]).

AIMS	TOOLS
Promote scientific performance and academic resilience	Process-oriented performance monitoring; Reflective performance assessment; Standardized evaluation procedures.
Standardized analysis of expectations and performance factors of all contributors	Focus on performance-determining processes; Systemic reflection tools.
Empowerment of self-efficacy, communication of expectancies and beliefs, and decisions to stay or leave	Psychological competence training; Coaching methods to strengthen self-efficacy, reflection, and communication.
Empowerment: Academic resilience by fostering mental strength, health, and well-being	Techniques from emotion, motivation, health, and neuropsychology; Trainings and interventions.
Identification and removal of performance obstacles	Regular transparent feedback, coaching measures, and supporting the meaningfulness of tasks.
Consideration of sociodemographic, gender, diversity, and cultural factors	Integration of sociodemographic variables; Intercultural coaching; Diversity-sensitive training.

### 5.3 Relationship between performance factors

Correlation analyses revealed significant relationships among participants’ evaluations of psychological performance factors, highlighting the importance of motivation, self-efficacy, and positive emotions as achievement emotions for academic success. Higher self-efficacy correlated with seeking support and exerting effort, while positive emotions correlated with self-confidence and performance motivation. Negative emotions were associated with worries about one’s academic performance. In line with the present findings, previous research has shown that positive emotions play a crucial role in enhancing self-confidence and performance motivation, whereas negative emotions are associated with performance-related worries [76]. Although achievement emotions [47], self-control, academic resilience [77], and emotional well-being appear to be important factors, they are still often overlooked in research on academic performance.

### 5.4 Perceived career barriers and reasons to leave academia

The present survey not only asked participants whether they plan to stay in or leave academia, but also examined specific barriers and reasons for their decisions. The semi-qualitative analysis of this data additionally identified prevalent daily obstacles that negatively affect performance, including time constraints, personal responsibilities, stress, financial worries, lack of motivation, and distractions. Moreover, the main reasons for why candidates thought about leaving academia clustered into four areas: a) financial issues and dissatisfaction with conditions; b) personal preference for the private sector; c) poor work-life balance and demanding hours, leading to d) low motivation and disengagement; e) administrative challenges, limited career opportunities, and social or cultural barriers linked to hierarchy, language, and cultural differences. These topics and issues were mentioned by PhD and postdoc candidates and provided by about half of the survey participants. This, however, does not mean that those who did not answer these open-ended questions did not perceive or experience any barriers, or were worried about telling. Looking at the opt-outs and missing (i.e., participants who did not provide reasons) revealed no gender- or stage-specific differences, suggesting that women or men, or PhDs or postdocs, did not significantly more often refrain from answering these questions.

In addition to dissatisfaction with financial working conditions, which were cited among the most pressing reasons to leave, the wish for more time, better work-life balance, and less pressure to better identify with academic work were cited as reasons not to pursue one’s academic career beyond the current goal. Of note, all the reasons provided align well with the concerns raised in educational reports, such as the OECD reports mentioned earlier [63–65], regarding attrition. Moreover, the benefits of working outside academia that influenced self-perceptions of career decisions should be investigated further, as the career landscape for PhDs has broadened and PhDs are making meaningful contributions across academia and other sectors [78].

The results underscore the relevance of addressing perceived challenges and barriers, as they affect young scientists’ self-regulation and career reflections. Fostering a sense of meaningful work [79] can be an effective strategy for these challenges. Engaging in work that aligns with personal values promotes a sense of purpose, which could reduce the likelihood of attrition. Additionally, teaching scientists about resilience [80], as suggested in Table 9 and [75], can promote self-regulatory skills for navigating the dynamics of the academic environment and for maintaining motivation and engagement in personal academic endeavors, thereby enhancing the capacity to function effectively under pressure and promoting long-term career satisfaction and stability.

### 5.5 Perceived supervision, early career development, and satisfaction with it

The participants’ answer about supervision was related to their experiences of being supervised and how they judged the regularity of the supervision, ranging from never (1) to daily (9). The results suggest that some respondents feel they do not receive regular supervision. Regarding supervision quality, only a few participants were fully dissatisfied. Only 26% believed supervisors need more support, and 34% said supervision programs for those being supervised should be more widely promoted and receive greater scientific recognition. Few candidates (PhD or postdoc) had experience with early-career tools like mentoring or coaching. The results support the notion that early-career candidates need greater literacy about the purpose and effectiveness of early-career development tools to benefit from them. Despite their importance, supervision roles and demands are often invisible to those supervised, yet supervisors’ desire for support and appropriate resources is strong [81,82].

## 6. Conclusion, limitations, and outlook

This approach examined young scientists’ beliefs and expectations about the core factors they consider most relevant to achieving their academic goals, as well as the barriers and obstacles, underscoring the relevance of these psychological factors as performance and health promoters and as career determinants. The survey found that for PhDs and postdocs, motivation, self-efficacy, effort, and confidence in their own skills were key factors for academic success and career achievement. Participants reported experiencing both positive and negative emotions simultaneously, with frustration being especially common, particularly among women. The choices to stay, leave, or remain unsure about one’s future career path were linked to differences in work motivation, engagement, sense of responsibility, beliefs about the effectiveness of support, and satisfaction.

### 6.1 Limitations and outlook

Although the present survey is novel, it has advantages and limitations that must be considered to avoid overgeneralization or speculative conclusions. The survey was advertised to postdocs and PhDs at various institutions across three countries (Germany, Austria, and Switzerland) who had similar educational backgrounds. Although career path fluidity is granted in these countries (e.g., allowing the options of doing a PhD in one country and starting a post-doc in one of the other two countries), generalization to other higher education systems globally should be avoided, as previous research has shown differences between countries and educational systems (see Introduction).

The sample size may also be a limitation. Although the survey included participants from three countries and various disciplines, rather than being confined to a single country or scientific domain, the sample was not equally balanced across the three countries (most participants were from Germany, fewer from Austria or Switzerland).

Moreover, women and men had equal opportunities to contribute, yet the observed gender imbalance limits statistical analysis. In particular, the gender distribution requires further exploration of the reasons for this imbalance, as discussed earlier in detail in the Discussion section. In addition, recruitment through platforms such as Prolific, an opt-in, financially compensated participant pool, may have advantages, but it may also introduce selection bias related to participant motivation, availability, or sample composition, which may limit the representativeness and generalizability of the findings.

Differences among early-career researchers may exist, notably regarding additional “stressors” such as acculturation stress, cultural differences, and traditional role models, as observed among international students [60]. In the present survey, students from countries other than the three target countries were not excluded from participation (9% of the sample), but presenting the survey in German might have limited participation. The advantages of German as a language choice are comprehensiveness, familiarity, and reduced misunderstandings of the items and questions among survey participants who are fluent in German. Research in affective neurolinguistics suggests that the language in which surveys are presented affects how participants self-disclose, how they express and remember emotionally laden events and situations, and how they respond to personally relevant content [83,84]. Thus, participants might respond more elaborately and precisely when they write in their strongest contextual language — especially in open-text fields. Regarding language aspects and choices in surveys, future research should investigate them more systematically, as they may affect survey results more than previously thought.

Participants included PhD candidates and postdocs, allowing cross-sectional comparisons. They could be working at a university, including a private university, or at a research institution, and be funded by others or by themselves. Future studies could benefit from a longitudinal design that examines transitions from university student to PhD and from PhD to postdoc, and also explores the data as a function of workplace environment (university, research, etc.). Because of its cross-sectional design and small sample size for these questions, the current approach cannot verify actual career trajectories or differences between working environments/conditions, leaving some questions open. Moreover, future studies could include supervisors and their expectations. A longitudinal survey approach would also verify the stability of differences observed between participants planning to stay in or leave academia, and between those who truly quit and those who wanted to stay but did not. It would also assess whether those who wanted to stay achieved their goals.

Future research should validate the survey results, both theoretically and empirically. As outlined in this manuscript, many theoretical frameworks exist (see Table 1). Replicating the present survey with significantly larger samples can take important first steps toward testing conceptual models and relationships among the psychological factors examined in the present survey.

Despite its limitations, the results of the present survey can inform the role of psychological factors in academia and why they should be included in career development strategies, enabling early-career researchers to identify with the project goals, their roles, and their development objectives. Using semi-quantitative surveys like the present one can enable future studies to identify the needs of PhD students, postdocs, and, if included, also supervisors. This could lead to tools that personalize support and improve scientific performance and well-being among researchers, and build resilience, motivation, and self-efficacy through appropriate supervisory and early-career measures, as suggested in Table 9.
